# Proximity of public elementary schools to major roads in Canadian urban areas

**DOI:** 10.1186/1476-072X-10-68

**Published:** 2011-12-21

**Authors:** Ofer Amram, Rebecca Abernethy, Michael Brauer, Hugh Davies, Ryan W Allen

**Affiliations:** 1 Department of Geography, Simon Fraser University, Burnaby, BC, Canada; 2 School of Population and Public Health, The University of British Columbia, Vancouver, BC, Canada; 3 Faculty of Health Sciences, Simon Fraser University, Burnaby, BC, Canada

## Abstract

**Background:**

Epidemiologic studies have linked exposure to traffic-generated air and noise pollution with a wide range of adverse health effects in children. Children spend a large portion of time at school, and both air pollution and noise are elevated in close proximity to roads, so school location may be an important determinant of exposure. No studies have yet examined the proximity of schools to major roads in Canadian cities.

**Methods:**

Data on public elementary schools in Canada's 10 most populous cities were obtained from online databases. School addresses were geocoded and proximity to the nearest major road, defined using a standardized national road classification scheme, was calculated for each school. Based on measurements of nitrogen oxide concentrations, ultrafine particle counts, and noise levels in three Canadian cities we conservatively defined distances < 75 m from major roads as the zone of primary interest. Census data at the city and neighborhood levels were used to evaluate relationships between school proximity to major roads, urban density, and indicators of socioeconomic status.

**Results:**

Addresses were obtained for 1,556 public elementary schools, 95% of which were successfully geocoded. Across all 10 cities, 16.3% of schools were located within 75 m of a major road, with wide variability between cities. Schools in neighborhoods with higher median income were less likely to be near major roads (OR per $20,000 increase: 0.81; 95% CI: 0.65, 1.00), while schools in densely populated neighborhoods were more frequently close to major roads (OR per 1,000 dwellings/km^2^: 1.07; 95% CI: 1.00, 1.16). Over 22% of schools in the lowest neighborhood income quintile were close to major roads, compared to 13% of schools in the highest income quintile.

**Conclusions:**

A substantial fraction of students at public elementary schools in Canada, particularly students attending schools in low income neighborhoods, may be exposed to elevated levels of air pollution and noise while at school. As a result, the locations of schools may negatively impact the healthy development and academic performance of a large number of Canadian children.

## Introduction

Motor vehicles are a major source of both air and noise pollution in communities. Epidemiologic studies have linked exposure to traffic-generated air pollution with a wide range of adverse effects in children including reduced lung function [1], decrements in lung growth [2], incident asthma [3], otitis media [4], and decreased cognitive function [5]. Chronic exposure to traffic noise among children has been linked with increased blood pressure [6], reduced sleep quality [7], and cognitive deficits [8].

For children, school is an important environment for exposure to traffic-related pollution due to the amount of time spent there [9]. According to the Canadian Human Activity Pattern Survey, children 11-17 years spend an average of 12% of time of their time at school, making it the second most common microenvironment, while for children < 11 years school is the 3^rd ^most important microenvironment, accounting for 6% of time on average [10]. Both noise and traffic-generated air pollutants such as diesel soot, ultrafine particles, oxides of nitrogen (NO_x_), and carbon monoxide are elevated within approximately 100-500 meters of major roadways [11-15], so the proximity of schools to major roads may be an important determinant of exposure. A study in the Netherlands found that for children attending a school within 100 m of a freeway, soot exposure was 30% higher and NO_x _exposure was 37% higher than among children attending a school in a background location [16]. Students attending schools close to major roads can be exposed to traffic-related air pollution even while indoors because outdoor pollution infiltrates into classrooms [17,18]. Inverse correlations between concentrations of traffic-related air pollution inside classrooms and distance to the nearest major road have been reported [19,20].

Several studies have quantified the distances from schools to major roads in the US [21-24], but no results for schools in other countries have been published. Here we present the results from an investigation of the roadway proximities of public elementary schools in the ten largest Canadian cities. Our objectives were to: 1) validate the use of roadway proximity as a surrogate for outdoor concentrations of traffic-generated air and noise pollution using measurements from previous field studies; 2) determine the proximities of public schools to major roads; and 3) explore urban characteristics and socio-economic indicators as correlates of schools' proximities to major roads.

## Methods

### Schools Data

Public elementary schools for the 10 most populated cities in Canada were chosen for this analysis as children in this age group may be particularly susceptible to the effects of environmental pollutants. Elementary schools were defined as those with students in kindergarten through grade 5 (schools with students in other grades were included if their enrollment also included students in grades of interest). We included only public schools due primarily to concerns about the quality and completeness of private school data in provincial databases. In addition, only about 6% of Canadian students attend private schools [25]. Cities from five provinces were included in the analysis. In Ontario: Toronto, Hamilton, Mississauga and Ottawa; in Quebec: Montreal and Quebec City; in Alberta: Calgary and Edmonton; in British Columbia: Vancouver; and in Manitoba: Winnipeg. For each city we only included schools within the municipality as defined by the census subdivision (i.e., we did not include schools in suburban communities). We included only urban areas primarily due to their higher levels of traffic-related air pollution and noise and concerns about geocoding accuracy in low density communities [26,27]. In addition, the majority of pollution measurements used to validate road proximity as a surrogate for pollution levels (described below) were collected in urban areas. To evaluate the sensitivity of our results to the exclusion of suburban communities, we randomly selected one census subdivision adjacent to each of five of our cities and geocoded the locations of schools in those five suburbs (the adjacent census subdivisions were Burlington, Brampton, Laval, Markham, and Richmond; these are adjacent to Hamilton, Mississagua, Montreal, Toronto, and Vancouver, respectively).

Relevant school attributes included the address, grade levels and type of school (public, private or other). As information regarding Canadian educational institutions is not centrally collected, the majority of this information resides with the provincial Ministries of Education (MoE). As a result, the availability and format of these data differ by province. Public school locations were collected using data available from the MoE websites for each province [see Additional file 1].

### School Geocoding and Road Proximity Calculations

We used the commercially available DMTI CanMap road network to identify road locations and attributes (DMTI Spatial, Markham, ON). This product covers Canada and divides roads into 6 categories. In our primary analysis we combined DMTI road categories 1 (expressways, usually four lanes), 2 (principal highways, which are multi-lane conduits for intracity traffic), 3 (secondary highways, which are typically thoroughfares with multiple lanes and large traffic capacity), and 4 (major roads, used for shorter trips within the city) into a single layer (henceforth "major roads") for analysis of school proximities [28]. GeoPinPoint (GPP) software, a product of DMTI, was used to geocode school addresses into latitude/longitude coordinates using a 10 m offset from the road's centerline. Designed for use within Canada, GPP uses the DMTI road network and allows for the geocoding of French-language addresses. In addition, it provides a summary output that details the number of schools successfully geocoded.

After geocoding, we calculated the distance from each school to the nearest major road using ArcGIS 9.2 (ESRI, Redland, CA). As a secondary analysis, we also quantified the distance from each school to the nearest expressway or principal highway (DMTI road categories 1 and 2) to allow for comparisons with previous studies in the US [21-23].

### Assessing Geocoding Accuracy

Because school buildings and grounds cover large areas, and because geocoding of schools can produce substantial location errors [27], we manually determined the locations of a subset of schools for comparison with our automated geocoding results. First, we selected schools with geocoded locations within 200 m of a major road (N = 533). From these we randomly selected 148 schools (10% of the 1,476 schools in the analysis) while requiring that at least five schools from each city be included. For each of these 148 schools, we then used satellite images from Google Maps to manually determine the coordinates of the point of the school building nearest to a major road and calculated the distance from that location to the nearest major road using ArcGIS. The major road proximities assessed by this manual method were considered the "true" distances for comparison with the distances estimated from the GPP geocoding procedure.

### Pollution Data

We used measurements from previous field sampling campaigns in Edmonton, Vancouver, and Winnipeg to validate the assumption that roadway proximity acts as a surrogate for concentrations of traffic-generated air pollution and noise. Nitrogen oxide (NO) concentrations were measured on a 2-week basis using passive Ogawa samplers at 50 locations each in Edmonton and Winnipeg [29] and 105 locations in Vancouver [30]. Locations were selected to cover the study areas and to capture a wide range of road proximities. Each location was monitored twice in different seasons, and the two measurements at each location were combined to estimate the long-term average concentration. Abernethy et al. [31] measured concentrations of ultrafine (< 0.1 μm diameter) particles, another traffic-generated air pollutant, for 1-hour periods at 80 of the NO monitoring locations in Vancouver using condensation particle counters (TSI CPC 3007, Shoreview, MN). Measurements were adjusted to account for temporal variation in ultrafine particle concentrations, and comparisons of measurements collected in different seasons suggest that 1-hour measurements represent long-term conditions. Davies et al. [32] measured 5-min equivalent continuous sound pressure levels (L_eq_) at the NO monitoring locations in Vancouver using a Larson Davis 870B sound level meter (Larson Davis, Depew, NY). We have previously shown a strong correlation between 5-min noise levels measured in different seasons [11], suggesting that these measurements are indicative of long-term noise levels. The locations of these NO, ultrafine particle, and noise measurements were recorded by field technicians using GPS. We used ArcGIS to calculate the distance from the measurement locations to the nearest major road, defined using the same DMTI data and road classification scheme as in the school proximity calculations.

### Correlates of Road Proximity

Data from the 2006 Canadian census were used to evaluate the relationship between schools' proximities to major roads and both dwelling density and socio-economic variables. Because we hypothesized that both city- and neighborhood-level characteristics might be correlated with proximity, we obtained data for the census subdivision (CSD) and census tract (CT) in which each school is located. CSD areas correspond to city boundaries, while CTs typically have populations between 2,500 and 8,000 and are useful proxies for neighborhoods in Canada [33]. Specific variables included dwelling density at both the city (CSD) and neighborhood (CT) levels as well as median income and percent of population without a high school diploma or equivalent at the neighborhood level [23]. Socio-economic variables were not considered at the city level because these variables are assumed to be meaningful primarily in the local context and may not be directly comparable between cities due to differences in costs of living or other factors. To account for clustering within cities and neighborhoods, we used mixed models with random intercepts at the city and neighborhood levels. We modeled school proximity as a binary proximity variable with 75 m cut point (PROC GLIMMIX, SAS v9.2) and also as a continuous variable (PROC MIXED). Although the influence of traffic-generated air pollution and noise extends well beyond 75 m [14], we chose this distance to be conservative, given geocoding errors and the relatively large areas of schools and playgrounds. To evaluate the sensitivity of model results to the choice of binary distance, we also modeled school proximity as a binary variable using 200 m cut point. Contrasts in predictor variables were scaled to roughly correspond to interquartile ranges (IQR) to allow for comparisons of effect sizes between variables.

## Results

Addresses were obtained for a total of 1,556 public elementary schools, 1,476 (94.9%) of which were successfully geocoded into a latitude/longitude location (Table 1). The geocoding success rate in individual cities ranged between 75.7% (in Calgary) and 100% (in four cities). Variables affecting geocoding success included addresses with no match in the road network and use of post office boxes as mailing addresses. The populations in the 10 cities included in this analysis ranged between approximately 490,000 in Quebec City and 2.5 million in Toronto [34]. The combined population of these 10 cities was approximately 9.5 million, or nearly one third of the total Canadian population.

**Table 1:** City characteristics and proximities of public elementary schools to major roads

City	City Population^a^	City Area (km^2^)^a^	City Population Density (persons/km^2^)^a^	City Dwelling Density (dwellings/km^2^)^a^	Median (IQR) School Neighborhood Dwelling Density (dwellings/km^2^)^b^	Median (IQR) School Neighborhood Income ($10,000)^b^	Median (IQR) School Neighborhood % Population Without HS Diploma (%)^b^	# of Schools Successfully Geocoded	Geocoding Success Rate (%)	Distance to the Nearest Highway or Major Road	
										
										Mean (± SD)	Median
Toronto	2,503,281	630	3,972	1,554	1,805 (1,774)	5.46 (2.11)	7.0 (6.5)	487	100.0	265 ± 197	240
Montreal	1,620,693	365	4,439	2,036	4,058 (3,780)	3.61 (0.86)	8.5 (6.1)	169	95.5	181 ± 156	156
Calgary	988,193	727	1,360	530	973 (527)	6.42 (2.37)	4.7 (4.6)	84	75.7	431 ± 275	366
Ottawa	812,129	2,778	292	116	910 (892)	7.76 (3.81)	3.5 (2.8)	116	100.0	346 ± 298	278
Edmonton	730,372	684	1,067	435	987 (498)	5.71 (2.22)	7.9 (6.3)	137	87.3	362 ± 193	346
Mississauga	668,549	289	2,317	745	1,185 (1,012)	7.51 (2.70)	5.6 (3.2)	103	100.0	445 ± 233	397
Winnipeg	633,451	464	1,365	563	1,162 (736)	4.93 (2.36)	7.8 (5.7)	163	97.0	402 ± 290	368
Vancouver	578,041	114	5,039	2,209	1,761 (1,185)	5.17 (1.23)	6.6 (7.6)	93	86.9	212 ± 153	191
Hamilton	504,559	1,117	452	174	1,152 (1,045)	5.87 (3.01)	7.7 (4.0)	98	100.0	278 ± 217	265
Quebec	491,142	454	1,081	502	3,166 (3,582)	3.37 (2.22)	6.5 (6.0)	26	81.3	257 ± 198	217
All	9,530,410	7,623	1,250	509	1,385 (1,515)	5.42 (2.73)	6.7 (6.2)	1,476	94.9	318 ± 221	282

^a^2006 census subdivision statistics.

^b^2006 census tract statistics.

IQR = interquartile range.

**Figure 1:**
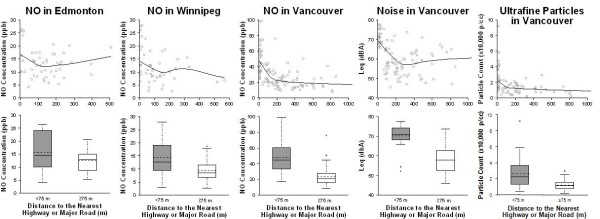
**Measured nitrogen oxide, ultrafine particles, and noise vs. distance to the nearest major road in three Canadian cities**. Lines in the upper plots are locally weighted regression curves fit to the data. Solid lines in boxplots represent medians; dashed lines represent means.

Pollution measurements in Edmonton, Vancouver, and Winnipeg were inversely correlated with the natural logarithm of distance to the nearest major road, with stronger correlations in Winnipeg (*r *= -0.44; p < 0.01) and Vancouver (*r *= -0.50 to -0.61; p < 0.01) than in Edmonton (*r *= -0.27; p = 0.06). Similar correlations were found when including only measurements within 200 m of a major road. Based on these measurements we conservatively defined 'near roads' as < 75 m (Figure 1). Mean (± SD) NO concentrations measured < 75 m from the nearest major road were greater than those measured ≥ 75 m in both Winnipeg (14.4 ± 7.3 ppb vs. 9.5 ± 4.2 ppb; 2-sample t-test p-value: < 0.01) and Vancouver (48.1 ± 20.3 ppb vs. 23.4 ± 11.3 ppb; p < 0.01). In Edmonton the difference was less pronounced (15.6 ± 7.4 ppb vs. 12.6 ± 4.1 ppb; p = 0.17). Ultrafine particles (26,000 ± 18,200 p/cc vs. 12,000 ± 6,200 p/cc; p < 0.01) and noise (70.2 ± 5.7 dBA vs. 57.9 ± 6.5 dBA; p < 0.01) in Vancouver were also significantly elevated within 75 m of major roads. In fact, in Vancouver the influence of major roads extended to approximately 200 m (Figure 1). Concentrations within 200 m of a major road were significantly higher than those ≥ 200 m for NO (39.7 ± 20.1 ppb vs. 20.6 ± 9.1 ppb; 2-sample t-test p-value: < 0.01), ultrafine particles (21,300 ± 16,300 p/cc vs. 11,700 ± 6,400 p/cc; p < 0.01), and noise (65.1 ± 8.2 dBA vs. 57.2 ± 6.6 dBA; p < 0.01).

**Figure 2:**
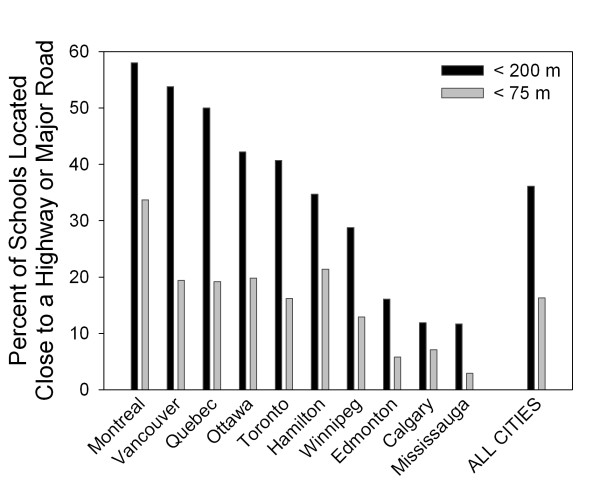
**Percent of public elementary schools that are located close to a highway or major road by city**.

Across all 10 cities, 16.3% of schools were located within 75 m of a major road (Figure 2). There was considerable variability between cities, ranging between 2.9% of schools in Mississauga, Ontario and 33.7% in Montreal, Quebec. Using a less conservative cut-off distance of 200 m, 36.1% of schools were located close to a major road, ranging between 11.7% of schools in Mississauga and 58.0% in Montreal (Figure 2). There was not a strong east-west gradient in school proximities by city. When considering only expressways or principal highways (DMTI road categories 1 and 2) to allow for comparisons with previous studies in the US, we found that 4.7% of schools were located within 200 m, ranging between 0% in both Calgary and Hamilton and 16.0% in Montreal.

Based on comparisons between five cities and communities adjacent to each, we found that a larger percentage of schools included in our analysis were located near major roads than schools in adjacent communities. In Hamilton, Mississauga, Montreal, Toronto, and Vancouver 18.7% of 950 schools were within 75 m of a major road, while in the five selected adjacent communities 9.3% of 236 schools were within 75 m. The cities all had higher percentages of proximate schools than their adjacent communities, with the exception of Mississauga, where the percentage of schools within 75 m of a major road (2.9%) was lower than the adjacent community of Brampton (4.1%).

**Figure 3:**
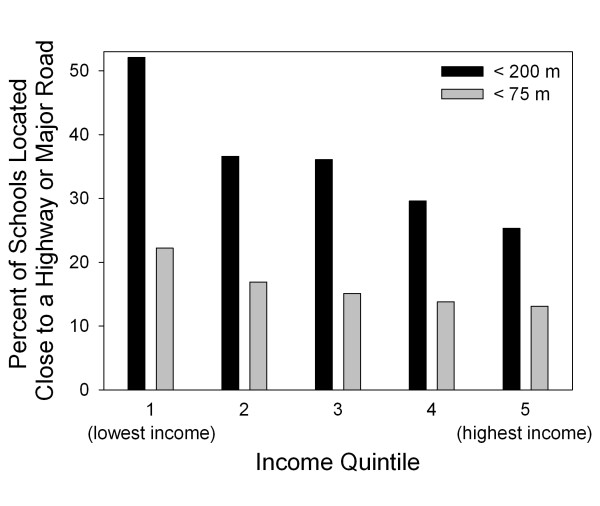
**Percent of public elementary schools that are located close to a highway or major road by city-specific quintile of median neighborhood-level income at the school location**.

When modeling schools' proximities to major roads as a binary variable (< 75 m or ≥ 75 m) we found that both higher neighborhood dwelling density (OR per 1,000 dwellings/km^2 ^increase: 1.07; 95% CI: 1.00, 1.16) and lower neighborhood median income (OR per $20,000 increase: 0.81; 95% CI: 0.65, 1.00) were associated with closer school proximity to the nearest major road (Table 2). Similar results were observed when schools were categorized as < 200 m or 200 m, and when school proximity was modeled as a continuous variable (Table 2). For example, for each $20,000 increase in median neighborhood income, schools were an average of 47 m (95% CI: 33, 61) further from major roads. The relationship between neighborhood median income and school proximity to roads is summarized in Figure 3, which shows the percent of schools in close proximity to major roads in each city-specific neighborhood-level income quintile. Of the schools located in the poorest neighborhoods in each city, more than 22% are < 75 m from a major road. In the highest income quintile only 13% of schools are within 75 m of a major road. The same relationship with neighborhood income was observed when close proximity was defined < 200 m (Figure 3).

**Table 2:** Results of multi-level models of school proximity to major roadways

Model Outcome Variable	Level Variable	Contrast^a^	Effect Estimate (95% CI)^b^
**School within 75 m of a highway or major road (binary)**	**City (Census Subdivision)**		
	Dwelling Density	1000 dwellings/km^2^	1.26 (0.71, 2.24)
	**Neighborhood (Census Tract)**		
	Dwelling Density	1000 dwellings/km^2^	1.07 (1.00, 1.16)
	Median Household Income	$20,000	0.81 (0.65, 1.00)
	% of Population Without HS Diploma	5%	0.95 (0.77, 1.18)

**School within 200 m of a highway or major road (binary)**	**City (Census Subdivision)**		
	Dwelling Density	1000 dwellings/km^2^	1.34 (0.75, 2.38)
	**Neighborhood (Census Tract)**		
	Dwelling Density	1000 dwellings/km^2^	1.19 (1.09, 1.29)
	Median Household Income	$20,000	0.74 (0.63, 0.89)
	% of Population Without HS Diploma	5%	1.08 (0.91, 1.28)

**Distance from school to the nearest highway or major road (m, continuous)**	**City (Census Subdivision)**		
	Dwelling Density	1000 dwellings/km^2^	-51.6 (-102, -1.3)
	**Neighborhood (Census Tract)**		
	Dwelling Density	1000 dwellings/km^2^	-10.3 (-17.0, -3.6)
	Median Household Income	$20,000	47.1 (32.8, 61.3)
	% of Population Without HS Diploma	5%	4.7 (-11.8, 21.2)

^a^Contrasts roughly correspond to interquartile ranges (see Table 1) to allow for comparisons of effect sizes across variables.

^b^For the binary outcome models the effect estimates are the odds ratios per variable contrast. For the continuous model the effect estimates are the changes in average school distance to the nearest highway or major road (in meters) per variable contrast.

For the 148 schools that were manually located, the median absolute value difference in estimated major road proximity between the GPP geocoded locations and the manually determined locations was 26 m (range: 0 - 244 m). In general, the automated geocoding procedure resulted in similar road proximity (median distance: 89 m; range: 1 - 200 m) as the manual procedure (median distance: 81 m; range: 6 - 301 m). When dichotomizing the 148 schools as < 75 m or ≥ 75 m from the nearest major road, 119 schools (80%) were placed in the same category by both methods (Table 3). Of the 71 schools automatically geolocated within 75 m of a major road by GPP, 79% were actually within 75 m of a major road, 87% were actually within 100 m, 90% were actually within 150 m, and 97% were actually within 200 m.

**Table 3:** Comparison between GeoPinPoint geocoding and manual locating for a random subset of schools

	Distances Based on Google Maps Geocodes			
Distances Based on GeoPinPoint Geocodes	Number (%) of Schools < 75 m	Number (%) of Schools 75 - 100 m	Number (%) of Schools > 100 m	Totals
Number (%) of Schools < 75 m	56	6	9	71
	(38%)	(4%)	(6%)	(48%)
	
Number (%) of Schools 75 - 100 m	5	1	2	8
	(3%)	(1%)	(1%)	(5%)
	
Number (%) of Schools > 100 m	9	9	51	69
	(6%)	(6%)	(34%)	(47%)
	
Totals	70	16	62	148
	(47%)	(11%)	(42%)	(100%)

## Discussion

We found that 16.3% of schools in Canada's 10 largest cities were located within 75 m of a highway or major road. To our knowledge this is the first such study outside of the US. Unlike previous studies of schools' proximities to major roads, we used measurement data to demonstrate a clear relationship between proximity to major roads and elevated levels of traffic-related noise and air pollution, and defined close proximity based on those measurements. A growing body of epidemiologic evidence links chronic exposure to traffic-related air pollution and noise with a wide range of health effects in children [1-6,13,35]. Thus, poorly sited schools may be placing a sizable fraction of Canadian public elementary school students at increased risk of adverse health effects. In addition, there is evidence that both noise [36,37] and air pollution [38] at schools may negatively affect academic performance.

Several studies have examined schools' proximities to major roads in the US. A study in California found that approximately 7.2% and 2.3% of public schools were within 150 m of medium (25,000-49,999 vehicles/day) and high traffic (≥ 50,000 vehicles/day) roads, respectively [23]. A similar study in Detroit found that 4.9% of schools were within 150 m of high traffic (≥ 50,000 vehicles/day) roads [22]. These results are generally consistent with our finding that 4.7% of schools were within 200 m of an expressway or principal highway. Most recently, Appatova et al. calculated roadway proximities for public schools in 9 major US cities and reported that 33% of schools were within 400 m of a major roadway (defined as federal interstate, US highway, or state highway) and nearly 12% were within 100 m [21].

Instead of using traffic volumes we defined major roads using the DMTI road classification scheme, which is standardized for roads across Canada. Although road categories are imperfect surrogates for pollution concentrations due to variability in traffic flows, vehicle types, and pollution emissions, our decision to define major roads using DMTI categories was supported by clear relationships with measured NO, ultrafine particles, and noise in 3 Canadian cities. A study in Vancouver found that roads in DMTI categories 1-4 (our definition of "major road") had mean daily traffic counts of 114,000, 21,000, 18,000, and 15,000 vehicles/day, respectively [28]. Our choice to define close proximity as only < 75 m from a major road for our primary analysis was more conservative than previous studies.

Our finding that neighborhood-level income correlated with school proximity to major roads has important environmental justice implications and is consistent with several previous studies indicating a relationship between socioeconomic status and environmental quality around schools. For example, Green et. al. [23] found that several indicators of lower socioeconomic status - including percentage of students receiving reduced-price meals at school, percentage of census tract population with income below poverty level, and percentage of census tract population with no high school diploma - were positively associated with traffic within 150 meters of schools in California. Similarly, Wu et al. [22] found that students attending schools near high traffic roads in and around Detroit were more likely to be ethnic minorities and to reside in a low-income area. A study in Sweden reported an inverse correlation between NO_2 _concentrations outside schools and neighborhood income [39]. Houston et al. reported that child care facilities in disadvantaged areas in California were more likely to be situated near busy roads than facilities in more affluent areas [40].

Unfortunately, our data did not enable us to investigate the chronology of school and road construction and neighborhood-level income changes, and more research is needed to understand the underlying causes of our findings. For example, it might be useful to explore whether low-income residents are drawn to neighborhoods with schools close to roads (e.g., due to lower housing prices), or if low-income neighborhoods are more likely to have schools and roads constructed in close proximity to one another (e.g., due to low-income residents having less influence on community decision-making) [41].

The relationship between neighborhood dwelling density and proximity demonstrates the challenge in balancing the health risks of environmental pollution with the potential benefits of urban living. Dwelling density and other indicators of urban "compactness" are often seen as desirable due to associations with increased physical activity [42,43] and decreased risks of obesity and associated morbidities [44-47]. However, our results and others' suggest that density may also lead to increases in exposure to environmental pollution. For example, Marshall et al. [48] found that Vancouver neighborhoods with a high walkability score (based on residential density, intersection density, retail floor area ratio, and land use mix) tended to also have relatively high levels of NO.

We did not find a strong east-west gradient in the fraction of schools located close to busy roads. This finding differs from the results of Appatova et al. [21] in the US. Their finding of a strong east-west gradient was driven primarily by schools on the "urban fringe" and they did not find a clear gradient for schools in urban centers. Thus, the lack of a clear gradient in our study may be due, in part, to our exclusion of suburban communities.

While this study provides the first assessment of schools' proximities to major roads outside of the US, some limitations should be noted. A 2007 study estimated that the median error for geocoded school addresses was 41 m, with larger errors in rural locations [27]. Location errors can be exacerbated by the large footprint of school buildings and surrounding playgrounds. In our assessment based on manually locating 10% of schools we found that the median error in estimated major road proximity was 26 m. However, the influence of location errors on our conclusions is minimized by our conservative choice of < 75 m as the distance of primary interest. In reality, the area of impact for vehicle emissions may extend out to 500 m depending on the specific pollutant [14]. We were encouraged that 87% of schools geocoded within 75 m a major road were actually within 100 m, while 90% were actually within 150 m. An additional benefit of our conservative definition of close proximity was that it reduced the influence of representing schools as points. As schools' sizes and layouts vary, the placement of classrooms and playgrounds in relation to roads can also affect students' exposures to traffic-related pollutants. Ideally, we would have overlaid the road network on a layer representing school footprints as polygons to calculate the portion of each school that is located in close proximity to a major road.

An additional limitation is that since schools data were not available from a single provider we relied on publicly available online data for this analysis, and this may have not captured all schools. In addition, data for private schools were not available or were incomplete for several cities, so this analysis included only public schools. However, while we may be missing some elementary schools in these cities, it seems unlikely that the roadway proximities of the missing schools would be systematically different from the included schools, and thus it is doubtful that our main findings and conclusions would be altered substantially by missing data. We only included schools within the census subdivision boundaries for major Canadian cities. Our sensitivity analysis suggested that, in general, schools in suburban communities were less frequently located in close proximity to major roads. Therefore, our results cannot be extrapolated outside of the cities included in this analysis. Nevertheless, since we included the 10 largest Canadian cities, which account for nearly one third of the Canadian population, our results apply to a large proportion of Canadian elementary students. Finally, we only considered the school environment, but other microenvironments and activities may make substantial contributions to the air pollution and noise exposures of school-aged children. For example, children can receive high exposures to some pollutants while commuting on diesel school buses [49], although exposures depend on a wide range of factors including emissions controls [50], fuels [51,52], and routes [52]. The relationship between school location and students' exposures is complicated by the fact that school location may affect accessibility and the amount of time that students spend in transit. In addition, attending a school located near a major road may also influence health by discouraging walking and cycling to school [53].

There are several possibilities for minimizing students' air pollution and noise exposures in and around schools. Concentrations of traffic-related air pollution can be reduced both by technical improvements that reduce per-vehicle emissions, such as improved engine efficiency, and urban planning/policy efforts that reduce automobile use, such as public transit enhancements and improvements in cycling infrastructure. New schools could be set back from major traffic corridors, and it may also be beneficial to orient the school facilities such that the outdoor playgrounds are located as far as possible from major roads [54]. For example, California State Bill 352 requires health risk assessments to be conducted for proposed school sites that are within 150 m of a busy roadway [55], while legislation in New Jersey (Assembly Bill 856, which was motivated by safety concerns and not environmental pollution) forbids the construction of new highway ramps within 300 m of an existing school [56]. The importance of traffic-related pollution in school siting decisions is also gaining recognition in Canada. For example, the British Columbia Ministry of Environment recommends that schools and other sensitive facilities be placed at least 150 m from roads with over 15,000 vehicles/day [57]. Given the small spatial scales over which traffic-related air pollutants and noise vary, shifting school locations by relatively small distances could result in substantial reductions in students' exposures, health risks, and impacts on academic performance.

There are also several potential strategies for reducing exposures at existing schools. As part of New York City's Asthma Free School Zone Project, Richmond-Bryant et al. [58] evaluated relationships between pollution concentrations and vehicle traffic and idling during school dismissal periods. They concluded that programs focused on school bus idling and redirecting school bus traffic could have small but measurable effects on diesel soot concentrations near schools. Some communities have implemented programs that limit outdoor activities during high outdoor air pollution days [59], but these programs are likely to be more effective for highly temporally variable pollutants like ozone than for traffic related pollutants, which are consistently elevated near roads. Another possible strategy is to modify school facilities. For example, the Port of Long Beach in California has created a "Schools and Related Sites Grant Program" in which schools and daycare facilities in close proximity to the Port may apply for funding to mitigate air pollution and noise impacts through improvements such as installing high efficiency particulate air (HEPA) filters in ventilation systems, replacing window and door seals, constructing sound barriers, and installing double glazed windows [60].

## Conclusion

We conducted the first assessment of schools' proximities to major roads outside of the US and found that 16% of public elementary schools were located within 75 m of highways or major roads. We conservatively chose 75 m as the distance of interest based on measurements of traffic-related air and noise pollution in 3 Canadian cities with different characteristics. There was considerable variability between cities in the percentage of schools located near roads, and distance to the nearest highway or major road was correlated with neighborhood income and inversely correlated with neighborhood dwelling density. In the lowest quintile of neighborhood income, 22% of schools were located within 75 m of a highway or major road. A substantial fraction of students at public elementary schools in Canada, particularly students attending schools in low income neighborhoods, may be exposed to elevated levels of air pollution and noise while at school. As a result, the school environment may negatively impact the academic performance and healthy development of a large number of Canadian children.

## Supplementary Material

Additional file 1 **Data sources for school locations and characteristics**. The table provides the websites used to obtain school addresses. Click here for file
