# Supplementary material for: Proximity of public elementary schools to major roads in Canadian urban areas
Source: Int J Health Geogr. 2011 Dec 21;10:68. doi: 10.1186/1476-072X-10-68 (PMC3283477; doi:10.1186/1476-072X-10-68)
Supplement: Additional file 1 — Data sources for school locations and characteristics. The table provides the websites used to obtain school addresses. [file 1476-072X-10-68-S1.PDF]

Additional file 1. Data sources for school locations and characteristics.

City	Link
Calgary	http://education.alberta.ca/apps/schoolsdir/
Edmonton	http://education.alberta.ca/apps/schoolsdir/
Hamilton	http://www.edu.gov.on.ca/eng/sift/index.asp
Mississauga	http://www.edu.gov.on.ca/eng/sift/index.asp
Montreal	http://www.mels.gouv.qc.ca/sections/publications/index.asp?page=statistiques
Ottawa	http://www.edu.gov.on.ca/eng/sift/index.asp
Quebec	http://www.mels.gouv.qc.ca/sections/publications/index.asp?page=statistiques
Toronto	http://www.edu.gov.on.ca/eng/sift/index.asp
Vancouver	http://www.bced.gov.bc.ca/apps/imcl/imclWeb/Home.do
Winnipeg	http://www.edu.gov.mb.ca/k12/schools
